# BK Virus Nephropathy Associated With Prolonged Immune Effector Cell–Associated Hematotoxicity Following Anti-CD19 CAR-T-Cell Therapy for Diffuse Large B-Cell Lymphoma in a Nontransplant Patient: A Case Report

**DOI:** 10.1016/j.xkme.2025.101143

**Published:** 2025-10-10

**Authors:** Naoto Okubo, Hanae Wakabayashi, Daisuke Honda, Yuuki Yoshioka, Tsubasa Shinomiya, Emiko Sakaida, Ryuji Ohashi, Katsuhiko Asanuma

**Affiliations:** 1 Department of Nephrology, Chiba University Hospital, Chiba, Japan; 2 Department of Hematology, Chiba University Hospital, Chiba, Japan; 3 Department of Integrated Diagnostic Pathology, Nippon Medical School, Tokyo, Japan

**Keywords:** BK virus, BK virus nephropathy, chimeric antigen receptor T-cell therapy, diffuse large B-cell lymphoma, immune effector cell–associated hematotoxicity

## Abstract

We report the case of a 67-year-old Japanese woman with diffuse large B-cell lymphoma who developed BK virus nephropathy (BKVN) following anti-CD19 chimeric antigen receptor T-cell (CAR-T) therapy. CAR-T therapy is an effective treatment for certain refractory hematologic malignancies; however, immune effector cell–associated hematotoxicity often presents as a clinical complication. Our patient developed progressive kidney dysfunction 6 months after CAR-T therapy in the setting of prolonged immune effector cell–associated hematotoxicity. BK viral DNA was detected in both the blood and urine, and kidney biopsy showed focal tubulointerstitial nephritis with simian virus 40 positivity in tubular epithelial cells, consistent with a BKVN diagnosis. Intravenous immunoglobulin (2 g/kg) was administered. The serum creatinine level and BK viral load continuously increased, possibly because of the delayed treatment. BKVN is a common complication in kidney transplant recipients but uncommon in nontransplant patients. As no established treatment for BKVN in nontransplant patients is currently available, early detection is crucial. To our knowledge, this is the first reported case of BKVN after CAR-T therapy. Routine monitoring for BK virus infection should be considered in patients experiencing prolonged myelosuppression after CAR-T therapy to enable early detection and prompt management of BKVN.

## Introduction

Chimeric antigen receptor T-cell (CAR-T) therapy has revolutionized the treatment of hematologic malignancies such as relapsed or refractory B-cell lymphoma and multiple myeloma, with response rates exceeding 50% in refractory diffuse large B-cell lymphoma.^1^, ^2^, ^3^, ^4^ More recently, its indications have expanded beyond oncology, with promising results in autoimmune diseases such as systemic lupus erythematosus and systemic sclerosis.^5^ Despite these advancements, CAR-T therapy is frequently associated with serious and potentially life-threatening toxicities, including immune effector cell–associated hematotoxicity (ICAHT), cytokine release syndrome, and immune effector cell–associated neurotoxicity syndrome.^6^

BK virus nephropathy (BKVN) is a complication primarily observed in immunosuppressed kidney transplant recipients but is rarely reported in nontransplant patients.^7^ BK virus (BKV) remains latent in renal tubular and urothelial cells and can reactivate when immune control is impaired, leading to tubulointerstitial nephritis.^8^ Reduction of immunosuppression is the main therapeutic approach against BKVN in transplant settings, but no standard treatment exists, and up to 15% of cases progress to end-stage kidney disease.^8^^,^^9^

To our knowledge, this is the first reported case of BKVN after anti-CD19 CAR-T therapy. The disease developed with prolonged myelosuppression in the absence of conventional immunosuppressive agents. As the use of CAR-T therapy is widespread, clinicians should be aware of this emerging complication.

## Case Report

The patient was a 67-year-old Japanese woman with no significant medical history except a 35-year smoking history (5 cigarettes/day). She was diagnosed with diffuse large B-cell lymphoma, not otherwise specified, Ann Arbor stage IV with multiple osteolytic lesions and generalized lymphadenopathy at 65 years. Her International Prognostic Index score was 3.

**Figure 1:**
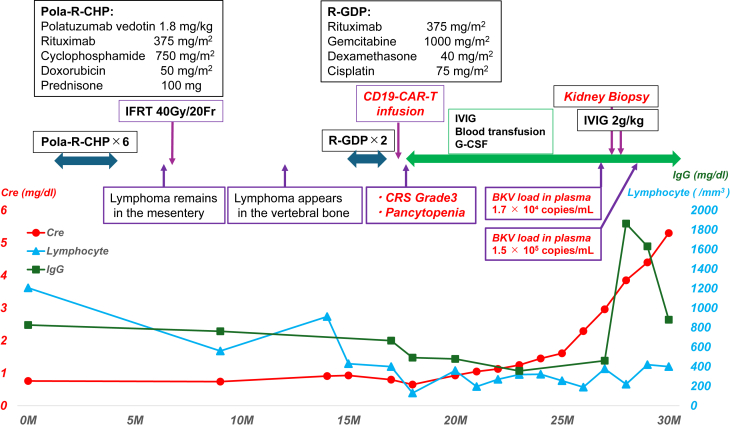
Patient’s clinical course. Clinical course: The month in which DLBCL was diagnosed was defined as 0M. The patient underwent 6 cycles of Pola-R-CHP therapy, followed by 2 cycles of R-GDP therapy for relapsed/refractory DLBCL. Despite treatment, the disease continued to progress. At 17M, the patient received anti-CD19 CAR-T infusion after conditioning with fludarabine and cyclophosphamide. Grade 3 CRS was observed on day 4, and pancytopenia persisted, requiring the administration of G-CSF and frequent blood transfusions. By 23M, serum creatinine levels began to increase. At 28M, BK virus DNA in plasma was 1.7 × 10^4^ copies/mL, prompting a kidney biopsy, which confirmed BK virus nephropathy. The patient was treated with intravenous immunoglobulin (IVIG, 2 g/kg for 1 week). Abbreviations: DLBCL, diffuse large B-cell lymphoma; CAR-T, chimeric antigen receptor T-cell; CRS, cytokine release syndrome; G-CSF, granulocyte colony-stimulating factor; IgG, immunoglobulin G.

She received 6 cycles of Pola-R-CHP therapy from 1-4 months. At 6 months, she received involved-field radiation therapy (40 Gy) targeting the intra-abdominal lesion. Despite treatment, the disease progressed. Between 15 and 16 months, she received 2 cycles of R-GDP therapy, resulting in severe cytopenia (Fig 1). Given that the disease was refractory, anti-CD19 CAR-T therapy was initiated at 17 months with a standard lymphodepletion regimen (fludarabine 25 mg/m^2^ and cyclophosphamide 500 mg/m^2^).

Her CAR-HEMATOTOX score was 0, indicating low risk. On day 4 postinfusion, the patient developed grade 3 cytokine release syndrome, successfully managed with tocilizumab (8 mg/kg × 2 doses) and dexamethasone (20 mg daily for 3 days, followed by 10 mg daily for 2 days). By day 15, she developed ICAHT, requiring granulocyte colony-stimulating factor and repeated transfusions. Regular fluorodeoxyglucose positron emission tomography scans every 6 months demonstrated complete metabolic remission, which was maintained throughout follow-up.

After CAR-T therapy, lymphocyte counts ranged between 100 and 400 cells/mm^3^, with CD4^+^ T-cell counts between 30 and 100 cells/μL and CD8^+^ T-cell counts between 50 and 277 cells/μL. Serum IgG levels were consistently below 500 mg/dL (Fig 1). Cytomegalovirus polymerase chain reaction was repeatedly negative.

**Figure 2:**
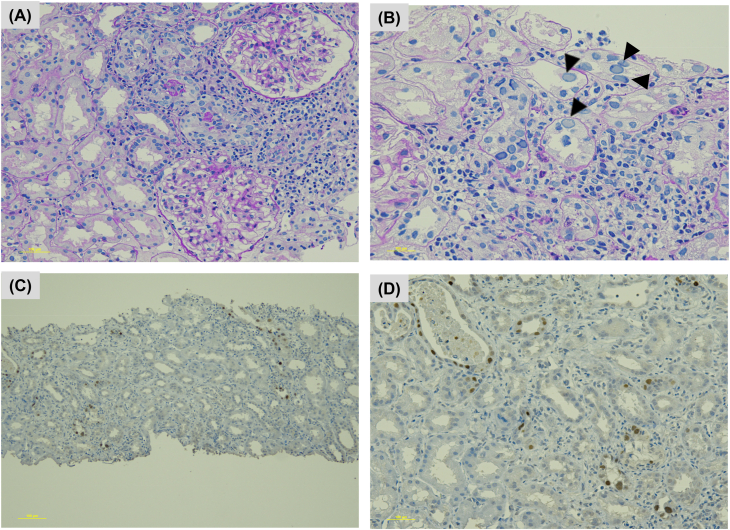
Histopathological examination and immunohistochemical staining. (A) The kidney biopsy revealed focally distributed tubulointerstitial nephritis, characterized by interstitial inflammation and tubular epithelial cell damage (periodic acid–Schiff staining, ×200). (B) Tubular epithelial cells were enlarged, exhibited ground-glass nuclei, and had nucleoli displaced to the nuclear periphery (arrowhead) (hematoxylin and eosin staining, ×400). (C) SV40 is positive in some tubular epithelial cells (positive rate: 1%-2%), using a mouse monoclonal anti–SV40 T antigen antibody (IHC SV40, ×40). (D) SV40 is positive in some tubular epithelial cells (positive rate: 1%-2%), using a mouse monoclonal anti–SV40 T antigen antibody (IHC SV40, ×200). Abbreviations: IHC, immunohistochemistry; SV40, simian virus 40.

At 23 months, her serum creatinine level, initially around 0.8 mg/dL, began increasing and reached 2.96 mg/dL by 28 months. Urinalysis results were negative for hematuria and proteinuria; however, urinary decoy cells were detected. At 28 months, quantitative polymerase chain reaction testing revealed BK viremia (blood BKV DNA load: 1.7 × 10^4^ copies/mL; urinary BKV DNA load: 5.0 × 10^7^ copies/mL). Kidney biopsy was performed (Fig 1). Only the renal cortex was sampled. Histopathological examination revealed focally distributed tubulointerstitial nephritis with marked tubular epithelial cell enlargement, ground-glass nuclear changes, and nuclear inclusion bodies in the affected areas (Fig 2A and B). Interstitial fibrosis was approximately 5%. Immunohistochemical staining for simian virus 40 was positive in the tubular epithelial cells (Fig 2C and D). These positive cells accounted for approximately 2% of all renal tubules and were identified using a mouse monoclonal anti-simian virus 40 T antigen antibody (Merck KGaA). Immunofluorescence staining was negative for IgG, IgA, IgM, C3, and C1q, and electron microscopy revealed no remarkable abnormalities. Based on these findings, the patient was diagnosed with BKVN, corresponding to polyomavirus nephropathy Class 2 (Banff lesion score: pvl2 and ci1), according to the 2019 Banff classification.

At 28 months, intravenous immunoglobulins (2 g/kg over 1 week) were administered based on a previous report.^10^ Despite treatment, kidney function continued to deteriorate. By month 30, the serum creatinine level had increased to 5.3 mg/dL, and the blood BK viral load had reached 1.5 × 10^5^ copies/mL, indicating a poor response to treatment (Fig 1).

## Discussion

ICAHT, characterized by cytopenia after CAR-T therapy, occurs in approximately 10%-40% of patients,^11^ and the CAR-HEMATOTOX score was developed to predict severe cytopenia. Although our patient was classified as low risk, she experienced prolonged and severe cytopenia. Previous studies have identified several clinical and disease-related factors, such as extensive marrow infiltration or severe cytokine release syndrome, as contributors to prolonged ICAHT.^11^^,^^12^ Anti-CD19 CAR-T cells typically decline within weeks to months after infusion.^5^ However, a subset of CAR-T cells persists as memory T cells, potentially remaining in the body for months to years. Persistence varies by CAR construct, with anti-CD19 CAR-T cells showing longer survival.^13^ Prolonged CAR-T cell persistence supports remission but increases the risk of opportunistic infection. Although not evaluated, long-term persistence may have contributed to ICAHT in this case.

In kidney transplant recipients, established risk factors for BKVN include lymphopenia (absolute lymphocyte count <500/μL), prolonged corticosteroid exposure (>7.5 mg/day prednisone), and intensive immunosuppression with agents such as mycophenolate mofetil and calcineurin inhibitors.^14^^,^^15^ In our patient, persistent lymphopenia was observed after CAR-T therapy. However, corticosteroid exposure was limited to short-term treatment for cytokine release syndrome, and she had never received mycophenolate mofetil or calcineurin inhibitors. These findings suggest that BKVN likely resulted from the combined effects of the cumulative chemotherapy toxicity and prolonged CAR-T–induced ICAHT, rather than conventional immunosuppressive regimens.

Similarly, hematopoietic stem cell transplantation is frequently accompanied by delayed immune reconstitution and shares pathophysiologic features with ICAHT following CAR-T therapy.^16^ In hematopoietic stem cell transplantation, BKV infection typically manifests as hemorrhagic cystitis, whereas biopsy-proven BKVN is rare. This rarity may reflect biopsy difficulty in thrombocytopenia, raising concerns about underdiagnosis.^17^ Previous studies in kidney transplant recipients have suggested that BKV-specific immunoglobulins may play a protective role against viral infection, particularly during the early stages of reactivation, although their efficacy diminishes once infection has advanced.^18^ In our case, persistent hypogammaglobulinemia likely contributed to the development of BKVN, and the delayed initiation of therapeutic intervention may have further diminished treatment efficacy. Collectively, these findings strongly suggest that patients undergoing CAR-T therapy are at increased risk of developing BKVN. Moreover, viral reactivation after CAR-T therapy is linked to delayed immune reconstitution. A narrative review notes that clinically significant cytomegalovirus infections occur in up to 10% of patients, and human herpesvirus-6 reactivation, which occasionally manifests as end-stage kidney disease, has also been documented.^19^

Currently, there are no well-established, evidence-based treatment guidelines for BKVN. In kidney transplant recipients, management typically involves reducing immunosuppressive agents and administering antiviral drugs (such as cidofovir) or intravenous immunoglobulins.^20^ In the present case, immunosuppressive agents were not used, and cidofovir is not approved in Japan; therefore, intravenous immunoglobulins were selected. Although intravenous immunoglobulins elevated the level of serum IgG, the copies of BKV DNA increased, and kidney function deteriorated with persistent urinary decoy cells. BKVN treatment response has been reported to correlate with initial BKV DNA levels, with poorer outcomes when viral loads exceed 1.0 × 10^4^ copies/mL.^21^ At the time of intervention, the viral load was 1.7 × 10^4^ copies/mL, which likely contributed to the poor response. Even in kidney transplant recipients, high viral loads are associated with poor outcomes despite a reduction in immunosuppression. In contrast, the immunosuppressive state induced by prolonged ICAHT after CAR-T therapy is often irreversible. Therefore, early intervention is essential. This case emphasizes the importance of initiating treatment while the BK load remains low in patients with prolonged ICAHT, where both immune recovery and therapeutic options are limited.

In this case, kidney function declined rapidly and progressed to end-stage kidney disease within 6 months, which was much faster than the typical 29-36 months observed in kidney transplant recipients.^22^ The patient was classified as polyomavirus nephropathy Class 2 according to the 2019 Banff classification, which is generally associated with only a modest increase in serum creatinine levels of around 1.0 mg/dL over 24 months. In comparison, polyomavirus nephropathy Class 3 cases typically show a greater increase of approximately 4.8 mg/dL during the same period.^23^ In the present case, the extent of fibrosis and the number of virus-infected cells were limited, which resulted in an underestimation of disease severity, likely because the biopsy specimen included only the renal cortex.

In light of these insights, monthly monitoring of plasma BKV DNA following screening protocols used in kidney transplant recipients^24^ should be considered for patients who develop lymphopenia (<500 cells/μL) and possess risk factors for prolonged myelosuppression after CAR-T therapy. Monitoring should be continued until ICAHT has resolved to enable early detection and timely intervention if BKVN develops.

In conclusion, BKVN should be recognized as a potential complication of CAR-T therapy. In patients with prolonged ICAHT, regular monitoring, early diagnosis, and timely initiation of available treatments are essential for improving the clinical outcomes of patients with BKVN.
